# Methane and nitrous oxide emissions in the rice-shrimp rotation system of the Vietnamese Mekong Delta

**DOI:** 10.1016/j.heliyon.2024.e35759

**Published:** 2024-08-08

**Authors:** Huynh Van Thao, Nguyen Van Cong, Le Thi Cam Nhung, Tran Hoang Kha, Huynh Cong Khanh, Le Van Dang, Nguyen Phuong Duy, Huynh Quoc Tinh, Trieu Nguyen Lan Vi, Nguyen Phuong Chi, Tran Sy Nam

**Affiliations:** a Department of Environmental Sciences, College of Environment and Natural Resources, Can Tho University, 3/2 street, Can Tho city, 900000, Viet Nam; b United Graduate School of Agricultural Science, Tokyo University of Agriculture and Technology, Fuchu city, 183-8538, Tokyo, Japan; c Western Highlands Agriculture and Forestry Science Institute, Buon Ma Thuot city, Dak Lak, 630000, Viet Nam; d Graduate School of Fisheries and Environmental Sciences, Nagasaki University, Nagasaki city, 852-8521, Japan; e College of Agriculture, Can Tho University, Can Tho city, 900000, Viet Nam; f WWF-Vietnam, No. 6, Lane 18, Nguyen Co Thach Street, Nam Tu Liem District, Ha Noi, 100000, Viet Nam

**Keywords:** Rice-shrimp rotation systems, Methane emission, Nitrous oxide emission, Greenhouse gas emissions, Vietnamese Mekong Delta

## Abstract

Rice-shrimp rotation systems are one of the widespread farming practices in the Vietnamese Mekong Delta coastal areas. However, greenhouse gas (GHG) emissions in the system have remained unclear. This study aimed to examine methane (CH_4_) and nitrous oxide (N_2_O) emissions from the system, including *(i)* land-based versus high-density polyethylene-lined (HDPE) nursery ponds and *(ii)* conventional versus improved grow-out ponds inoculated with effective microorganisms (EM) bioproducts. The results showed that CH_4_ flux in land-based and HDPE-lined nursery ponds were 1.04 and 0.25 mgCH_4_ m^−2^ h^−1^, respectively, while the N_2_O flux was 8.37 and 6.62 μgN_2_O m^−2^ h^−1^, respectively. Global warming potential (GWP) from land-based nursery ponds (18.3 g CO_2eq_ m^−2^) was approximately 3 folds higher than that of the HDPE-lined nursery pond (6.1 g CO_2eq_ m^−2^). Similarly, the mean CH_4_ and N_2_O fluxes were 15.84 mg CH_4_ m^−2^ h^−1^ and 7.17 μg N_2_O m^−2^ h^−1^ for the conventional ponds, and 10.51 mg CH_4_ m^−2^ h^−1^ and 7.72 μg N_2_O m^−2^ h^−1^ for the improved grow-out ponds. Conventional practices (2388 g CO_2eq_ m^−2^) had a higher 1.5-fold GWP compared to the improved grow-out pond (1635 g CO_2eq_ m^−2^). The continuation of the land-based nursery pond and conventional aquacultural farming practices increase CH_4_ emission and GWP, while applying HDPE-lined nursery ponds combined with improved grow-out ponds could be a promising approach for reducing GHG emissions in rice-shrimp rotation systems. This study recommends further works in the rice-shrimp rotation systems, including (i) an examination of the effects of remaining rice stubbles in the platform on the availability of TOC levels and GHG emissions and (ii) ameliorating dissolved oxygen (DO) concentration on the effectiveness of GHG emission reduction.

## 1 Introduction

Vietnamese Mekong Delta (VMD), located in Southern Vietnam and downstream of the Mekong River, is strategically positioned to develop agriculture production activities, especially rice cultivation and aquaculture practices. The VMD's annual rice production area is around 3.8 million ha, while the aquaculture area is approximately 1.9 million ha [1]. Although the VMD's area accounts for approximately 12 % of the total national area, rice production yearly produces more than 50 % of the total national rice productivity [[2], [3], [4]]. Furthermore, fishery production in the VMD also outlines ∼56 % of the productivity of the whole country [1]. Rice production is mainly centralised in localities where the freshwater source is easily accessible, whereas aquacultural production also pays attention to the coastal areas [5]. Intersectional or alternative areas between freshwater and seawater have recently involved sustainable rotation systems between agriculture and aquaculture, like rice-shrimp/fish rotation models or shrimp-crab co-culture. The VMD has 7 provinces in the coastal areas comprising Tien Giang, Ben Tre, Tra Vinh, Soc Trang, Bac Lieu, Ca Mau and Kien Giang, where diversifying farming practices are identified. The rice-shrimp rotation system is considered to be the best farming practice that has been practiced for over 22 years [6].

The VMD belongs to the tropical area; climatic conditions are divided into dry (November–April) and wet (May–October) seasons. Rice is cultivated in the wet season (September–November) when freshwater or rainfed are available, while in the dry season, saline intrusion raises the levels of salinity in the rivers/canals; rice cultivation might, therefore, struggle with high salinity [7]. As a result, aquacultural farms (shrimp, fish, and crab) have been encouraged as a primary livelihood activity under salinisation from February – July [6]. Among the mentioned provinces, Bac Lieu province shares a large area of rice-shrimp/crab rotation systems. The updated area of the rice-shrimp rotation system in the province is ∼39000 ha (expected to extend up to 43000 ha in 2025), of which the rice-shrimp area of Hong Dan and Phuoc Long districts is responsible for 25460 ha (0.35 million ton shrimp productivity) [8].

Shrimp and mud crab seeds are provided by local hatcheries, which are under strict quality control. Typically, the seed is directly stocked in their grow-out ponds. For each aquacultural crop, stocking can be executed from 2 to 3 times for shrimp and 1–2 times for crab. Harvest is ordinarily carried out using local net traps after stocking for around three months and prolonged till August [6]. The farmed practice has applied no commercial aquaculture feeds that utilise nature-based sources and the balance of its aquatic ecosystem. Therefore, the model is currently receiving much more attention for its adaptability and sustainability. However, direct stocking can cause unpredictable shrimp-cultured risks, so shrimp productivity gained is lower than expected yields [9]. Since 2021, World Wide Fund for 10.13039/501100020487Nature in Vietnam (WWF-VN) has rolled out better solutions for reducing environmental risks and expecting higher shrimp productivity throughout the promotion of a 20-day stage in nursery ponds prior to stocking into the grow-out pond. In addition, the suggestion includes *(i)* the application of co-incubation of effective microorganism (EM) bioproducts (*Saccharomyces cerevisiae* (10^9^ CFU kg^−1^), *Bacillus licheniformis* (10^9^ CFU kg^−1^), *Bacillus subtillis* (10^9^ CFU kg^−1^), enzyme amylase, protease, lipase, and necessary nutrients) with molasses and rice bran powder (co-incubated inoculum); and *(ii)* crushed-boiled fish as an organic feed source for shrimp [10]. The improved model is currently employed as pilot scales at various local farms in Bac Lieu province.

The sustainability of executing models is not only interested in shrimp productivity and offered economic efficacy for farm owners but also considered in the expressions of GHG emissions. Several studies have pointed out that GHG emissions from aquaculture ponds need to be considered as further important concerns [11,12]. The CH_4_ and N_2_O emissions are major concerns in aquaculture ponds, while CO_2_ could be relieved as it is returned to organic matter diagenesis, involved in carbon cycles, or fixed in its aquatic ecosystems [13]. If primary production surpasses CO_2_ generation in the sediment and water column, aquaculture ponds exhibit a crucial CO_2_-sink role [14]. In fact, it is evident that annual CO_2_ and CH_4_ fluxes in coastal earthen ponds were obtained by −18.4 and 22.6 mg m^−2^ h^−1^, respectively [15]. Likewise, Yang et al. [16] also show that the average fluxes of CO_2_, and CH_4_ of mixed shrimp and fish aquaculture ponds were −60.5 mgCO_2_ m^−2^ h^−1^, and 1.65 mgCH_4_ m^−2^ h^−1^, respectively, reminding that shrimp ponds are activated as a CO_2_ storage, and observed CH_4_ emission sources.

Moreover, the high availability of nitrogen-containing nutrients is one of the primary challenges in aquaculture ponds. Ammonium (NH_4_^+^) and nitrate (NO_3_^−^) ions can act as contributor materials for nitrification and denitrification processes that produce N_2_O from soil and water column [17]. The application of nitrogen-containing fertilizers could stimulate N_2_O production, resulting in increasing GHG emissions [18]. However, in China's rice-fish/shrimp co-culture system, Li et al. [11] recently revealed that N_2_O emissions were reduced by approximately 86–108 %, indicating a better understanding of GHG-emission reduction patterns. In shrimp ponds, N_2_O-related emissions come not only from material decompositions of the microbial community but also are generated from the intestinal tract of cultured species, contributing to overall GHG emissions in the ponds [19]. Therefore, the evaluation of GHG emissions from rice-shrimp systems has generated considerable interest, especially rice-shrimp rotation systems in the VMD, where GHG-emissions patterns have remained unclear.

Rice-shrimp rotation systems in the VMD apply no commercial feed sources while stocking shrimp. However, the availability of organic matter, particularly carbon sources from rice biomass in preceding crop, has persisted in the fields. Shrimp farms take advantage of rice stubbles as a natural feed source for aquaculture objects. The continuous immersion could generate anaerobic conditions that facilitate vigorous methanogenesis, resulting in higher CH_4_ emissions [20,21]. In contrast, CH_4_ and N_2_O oxidations also occurred aerobically at the surface of the sediment and in the oxic/anoxic water columns [20,22,23]. Moreover, the fluctuation of the water column in aquaculture ponds transits between aerobic and anaerobic conditions, of which aerobic decomposition promotes N_2_O emissions while the process slows methanogenesis [24]. Thus, stocking shrimp in the rice-shrimp rotation systems raised major concerns concerning the proliferation of GHG emissions.

The comparison of carbon and nutrient budgets in shrimp grow-out ponds of the rice-shrimp rotation system has been revealed by Dien et al. [7]. However, CH_4_ and N_2_O emissions from rice-shrimp ponds have remained ambiguous in the rice-shrimp rotation systems. In addition, the relationship between environmental variables and GHG emissions in aquaculture ponds can reveal the main factors contributing to the magnitude of GHG emissions that need to be taken into consideration for further effective solutions. Also, the encouragement of a 20-day stage application with a nursery pond was suggested, and the co-incubated inoculum for aquaculture ponds recommended by WWF-Vietnam in the context of GHG emissions has not been clarified. The clarification of CH_4_ and N_2_O patterns for nursery ponds and grow-out ponds and the provision of emission factors are the main motivations of this work. We, therefore, aimed to examine the CH_4_ and N_2_O emissions and provide emission factors for *(i)* the land-based and HDPE-lined nursery ponds and *(ii)* the conventional and improved grow-out ponds for rice-shrimp/crab rotation systems in Bac Lieu province of the VMD. The main contribution of this work is the GHG-emission reduction using co-incubation of EM in shrimp aquaculture and providing CH_4_ and N_2_O emission factors for data inventory in the rice-shrimp rotations systems under the context of the VMD.

## 2 Materials and methods

### 2.1 Study sites

**Fig. 1:**
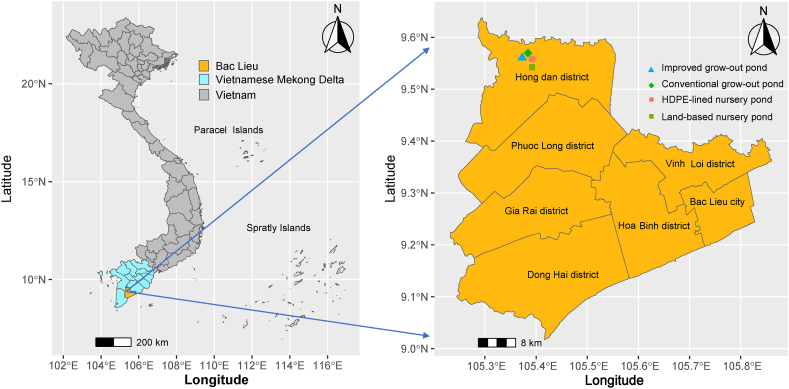
Map of study sites.

The experiment was conducted at three farmer's farms in Vinh Loc commune, Hong Dan district, Bac Lieu city, Vietnam (Fig. 1). The study area is a low-lying topographic (0.2–0.8 m) influenced by the semi-diurnal regime of the East Sea and West Sea, of which the East Sea plays a decisive role in the area's tide. The climate belongs to the tropical monsoon area. The average temperature varies between 24.9 and 29.8 °C over the years. The rainfall averages approximately 1874.4 mm; 90 % of the precipitation concentrates in the Wet season. The duration of the sunshine is around 6.6 h per day. The mean humidity is around 85 %. The location pertains to light salinisation. The rice-shrimp rotation systems are supposedly pertinent for the aquacultural and agricultural activities of the locality. Characteristics of soil physicochemical properties from 0 to 20 cm are as follows: sand, 2.3 %; silt, 45.1 %; clay, 52.6 %; K_sat_, 42.6 mm h^−1^; OC (organic carbon), 2.39 %; CEC (cation exchange capacity), 18.7 cmolc^+^ kg^−1^ [6]. The area is one of the representative aquaculture regions to execute the rice-shrimp rotation system of the VMD. Rice-shrimp rotation systems have been developing for more than 20 years. The vital species cultured in this system include *(i)* shrimp (*Penaeus monodon*) and *(ii)* mud crab (*Scylla paramamosain or S. olivacea*). Yearly, shrimp culture is launched from January to July (based on the lunar calendar), while rice crops typically start from August to November [6,7].

### 2.2 Shrimp post-larvae

Conventionally, shrimp larvae from local hatcheries are directly released in the grow-out ponds. However, the approach has been recently transformed toward a better innovation suggested by WWF-Vietnam that shrimp was nurtured through a ∼20-day period in nursery ponds to adapt to on-site environmental conditions before stocking [10]. In addition, EM bioproducts were co-incubated with molasses and rice bran powder was applied during the period. The nursing expectation was to reduce environmental risks/shocks and mortality/morbidity rates of shrimp larvae, causing further ineffectiveness of shrimp-achieved yield.

### 2.3 Experimental design

Sampling of GHG emissions was conducted in *(i)* land-based and nursery HDPE-lined ponds and *(ii)* conventional and improved grow-out ponds. A nursery pond was built to nurture shrimp larvae to an expected size before stocking. It is suggested that the stage was prolonged for ∼20-day stage. Then, the fostered shrimp was stocked directly into the grow-out ponds. Co-incubation was performed to provide the organic-derived feeds and toxicological-related risk. The co-incubated mixture was applied to both nursery ponds and only improved grow-out ponds.

#### 2.3.1 Nursery pond management

There were two types of nursery ponds applied, including *(i)* land-based nursery pond (Lb-N pond) (Fig. S1a) and *(ii)* HDPE-lined nursery pond (HDPE-N pond) (Fig. S1b). This study evaluated the CH_4_ and N_2_O emissions from the two nursery ponds. The area of the land-based pond was 150 m^2^, while the area of the HDPE-lined pond was 80 m^2^ (Table S1). The water-level depth of the land-based and HDPE-lined ponds was 1.5 m and 0.8 m, respectively. The Gold Key Aquatic Seed Biological Solution Co. Ltd provided the shrimp larvae, a reliable local hatchery that contributed a wide range of quality shrimp larvae in the region. The average density of stocking shrimp was applied at ∼400 shrimp m^−2^ for the HDPE-lined pond and ∼160 shrimp m^−2^ for the land-based pond. Nurturing times of shrimp were 20 days for the land-based and HDPE-lined ponds, respectively. The weight obtained from 4000 to 4700 shrimp kg^−1^ was stocked to the grow-out ponds. Co-incubated inoculation was disseminated into the nursery ponds every 3 days. In the HDPE-lined pond, the aeration process was performed continuously, and shrimp wastewater was eliminated every 3 days.

#### 2.3.2 Grow-out pond management

**Fig. 2:**
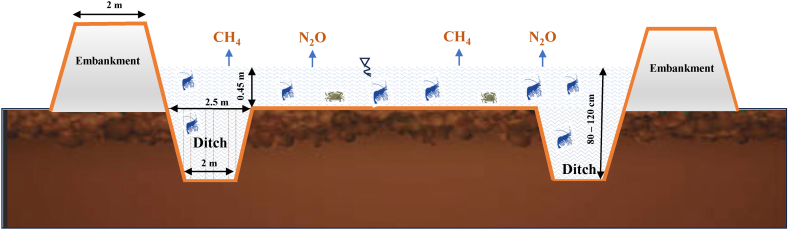
Transect of the rice-shrimp/crab rotation system.

The evaluation of GHG emissions for two grow-out ponds was performed on the *(i)* traditional rice-shrimp rotation system (without application nursery ponds) (CGo) and *(ii)* improved rice-shrimp rotation system (IGo) recommended by WWF-VN [10]. The area of the experimentally traditional and improved systems was 0.92 and 1.5 ha, respectively (Fig. S2). The main technical configuration of the rice-shrimp rotation system includes *(i)* a rice-shrimp platform and *(ii)* a shrimp-growing ditch (Fig. 2). The system was surrounded by a ∼2-m earthen embankment. The depth of water in the ditch and platform during stocking varied from 80 to 120 cm and 0 to 45 cm, respectively. The area proportion of ditch accounted for approximately 24.0 % and 18.3 % for conventional and improved grow-out ponds, respectively.

The shrimp stocking farms were previously growing rice. The rice harvest was done on January 6th, 2023. After harvesting, rice stubble has remained ∼10 cm onto the fields. Also, rice straw was removed from the farms using a round straw baler. Lime (Ca(OH)_2_) was applied on the field with 85.6 kg ha^−1^ and 85.9 kg ha^−1^ to improve pH for improved and conventional farms, respectively. Afterward, the field was reflooded with brackish water for a 4-day. The cycle was repeated three times to increase soil pH and clean up the farm. After the final reirrigation, brackish water was supplied from interior canals approximately ∼45 cm from the platform to prepare stoking shrimp.

The unnurtured and nurtured shrimps were released 5 and 3 times in the conventional and improved grow-out ponds, respectively. Furthermore, mud crab seeds provided by a local hatchery were released at once for an improved model and twice the traditional model as a supplementary object into the grow-out ponds. The density of shrimp and crab is shown in Table S2.

Co-incubated inoculum was applied to the improved farm every 10 days during cultivating times (164 L ha^−1^ per time). Water exchange was not performed frequently. It was applied only 2 times during stocking, which exchanged by 60–80 % of the total volume (Table S2). The input water was pumped directly from an adjacent canal. Fertiliser was also applied to both farms to accelerate the development of valuable algae known as natural foods for shrimp and aquatic species. However, the improved model applied organic fertilisers, while the conventional model. The total amount of chemical fertiliser and organic fertiliser farms applied for conventional and improved ponds is shown in Table S2.

### 2.4 Measurements

Air samples were collected weekly on the nursery ponds from April 28th – May 11th, 2023. In total, four sampling times were depleted for land-based and HDPE-lined nursery ponds. Nursery stages were done for a 19-day stage. Air samples were collected from grow-out ponds from April 28th to September 19th every 2 weeks, followed by Ma et al. [25]. Sample collection was carried out simultaneously at three sites of these farms. A floating chamber (0.6 m (length) x 0.4 m (wide) x 0.5 m (height)) was used to take air samples emitted from the nursery and grow-out ponds [11]. The floating chamber was made from a transparent acrylic material equipped with a gas-collecting port, a thermometer, a circulating fan, and a pressure bag (Fig. S3). The chamber was placed on the water's surface to collect air emitted over time. Gas samples were collected at 3 sites for each nursery (land-based and HDPE-lined ponds) and grow-out ponds (conventional and improved ponds). The collection was done after chamber closure at 1, 15 and 30 min. The change of air temperature and pressure inside chambers was recorded simultaneously. Also, temperature, pH, Eh, salinity and EC were detected by a portable meter (TOA-DKK cooperation, MM-41DP, Tokyo, Japan) with compatible electrodes. DO was also measured using a portable meter (TOA-DKK cooperation, DO-31P, Tokyo, Japan).

Water samples were also collected to analyse TOC, TN, TAN, NO_2_^−^, NO_3_^−^, and PO_4_^3−^. TAN, NO_2_^−^, NO_3_^−^, and PO_4_^3−^ were analysed using flow injection analyzer (FIALYZER-100, FIA lab, USA). TOC and TN were analysed simultaneously using a total nitrogen unit for TNML TOC-L Series (TOC-L CPH, Shimadzu) with Autosampler ASI-L. The concentrations of CH_4_ and N_2_O are analysed with a gas chromatograph (8610C, SRI Instruments, CA, USA) equipped with a flame ionisation detector (FID) and an electron capture detector (ECD) for the analysis of CH_4_ and N_2_O, respectively.

The hourly CH_4_ and N_2_O fluxes were calculated from the linear slope of each gas concentration over time [26]. The global warming potential (GWP) of combined CH_4_ and N_2_O emissions was calculated using the GWP from the Intergovernmental Panel on Climate Change (IPCC) for a 100-year time horizon, including climate-carbon feedbacks (34 for CH_4_ and 298 for N_2_O) [27].

Gas fluxes of nursery ponds (an HDPE-lined pond and a land-based pond) and grow-out ponds (conventional and nursey-pond applied models) were calculated using the following Equation (1):

(1) *F* = ρ x (V/A) x (*Δc/Δt*) x [273/(273+T)] x *P*/760

In which: where F is the ﬂux (mg CH_4_ m^−2^ h^−1^ or μg N_2_O m^−2^ h^−1^), ρ is the gas density (ρCH_4_ = 0.717 kg m^−3^ and ρN_2_O = 1.977 kg m^−3^ at 273 K and 760 mm Hg), V is the volume of the chamber (in m^3^), A is the cross-sectional area of the chamber (m^2^), Δc/Δt is the change in the gas concentration inside the chamber as a function of time, T is the average air temperature inside the chamber before and after gas sampling (in °C), 273 is the correction factor between °C and K, and P is the air pressure (in mm Hg). The value of P was the pressure recorded inside the chamber.

Based on the measurements of the gas fluxes, the annual cumulative fluxes of CH_4_ and N_2_O were calculated using Equation (2) [15]:

(2) Area emission (AE) = ∑ MF_i_ × D_i_ × 24hr

where AE is the emission (g CH_4_ m^−2^ or mg N_2_O m^−2^), MF_i_ is the mean CH_4_ or N_2_O flux in the ith month of the year (mg CH_4_ m^−2^ h^−1^ or μg N_2_O m^−2^ h^−1^), and D_i_ is the total number of days in the ith month.

The global warming potential was calculated using Equation (3) as follows:

(3) Global warming potential (GWP) = CH_4_ emission x 34 + N_2_O emission x 298

Where GWP (g CO_2eq_ m^−2^) is the cumulative amount of GHG emitted from the water surface of these ponds (g CO_2eq_ m^−2^), CH_4_ emission is the cumulative amount of CH_4_ emitted from ponds (g CH_4_ m^−2^), N_2_O emissions is the cumulative N_2_O emitted from ponds (in gN_2_O m^−2^).

### 2.5 Data processing

Comparison of gas fluxes or environmental variables between the conventional and advanced models uses the Independent Sample T-Test at a confidence level of 95 %. Principle component analysis (PCA) was a method that could be used for identifying important factors as well as to explain the relationship between variables and observations [28,29]. In this study, the PCA was used to identify key environmental factors contributing to the systems' GHG emissions. The Pearson correlation coefficient was employed to assess the relationship between variables [28,30]. Generalised Linear Models (GLMs) were performed to establish the relationship between affected factors according to Pearson scores and methane emission. Data were analysed using the RStudio software package (R Foundation for Statistical Computing, R version 4.2.3 for Windows).

## 3 Results

### 3.1 Nursery ponds

#### 3.1.1 Auxiliary environmental variation

**Fig. 3:**
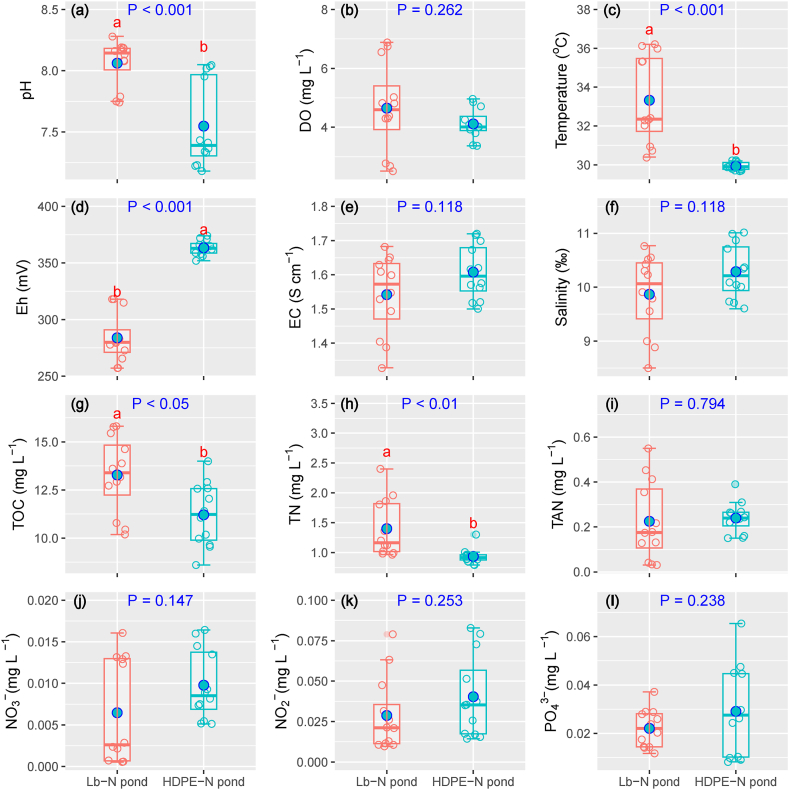
Surface water physicochemical properties of nursery ponds. Lb-N pond: land-based nursery pond; HDPE-N pond: HDPE-lined nursery pond. Different letters in each subfigure indicate significance at P = 0.05 by Independent Samples T-Test.

The physicochemical properties of water in Lb-N and HDPE-lined nursery ponds are clearly shown in Fig. 3. A higher pH value was identified in the HDPE-lined pond compared to the Lb-N pond (P < 0.001). Temperature values in the Lb-N ponds were higher than that of HDPE-N ponds (P < 0.001). Higher Eh values were observed in the HDPE-N pond than in the Lb-N pond (P < 0.001). EC and salinity shared the similarity of the trends with higher fluctuation to be identified in the Lb-N pond. TOC and TN concentrations were higher in Lb-N ponds than the HDPE-N pond (P < 0.05, and P < 0.01, respectively). The contents of DO, EC, salinity, TAN, NO_3_^−^, NO_2_^−^, and PO_4_^3−^ were insignificant differences between ponds.

#### 3.1.2 Variation in CH_4_ and N_2_O fluxes

**Fig. 4:**
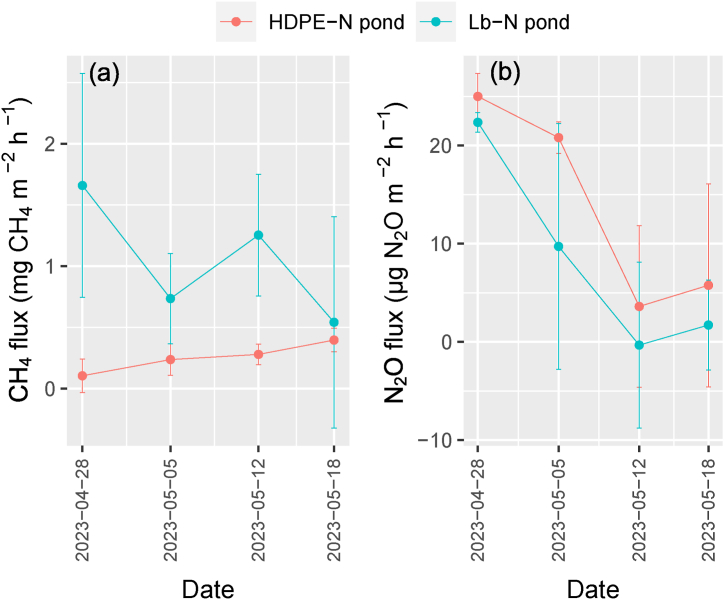
CH_4_ and N_2_O emissions from land-based and HEPD-lined nursery ponds. Lb-N pond: land-based nursery pond; HDPE-N pond: HDPE-lined nursery pond. Date format: yyyy/mm/dd.

The dynamics of CH_4_ during nursing shrimp in land-based and HDPE-lined nursery ponds are shown in Fig. 4a. CH_4_ emission during the nursing period for HDPE-N and Lb-N ponds fluctuated in the range of −0.05 – 0.50 mg CH_4_ m^−2^ h^−1^ and -0.28 – 2.66 mg CH_4_ m^−2^ h^−1^, respectively. The mean values of the CH_4_ flux in the HDPE-N and Lb-N ponds were 0.25 and 1.04 mg CH_4_ m^−2^ h^−1^, respectively (Table 1). Higher flux (0.79 mg CH_4_ m^−2^ h^−1^) was unveiled in the Lb-N ponds (P < 0.05). The highest CH_4_ peaks were detected in the Lb-N ponds, and the trend of variations was complex, while the CH_4_ emission patterns in the HDPE-N pond gradually increased until the nursery-stage termination. Moreover, the variation (standard deviation) of CH_4_ flux in the HDPE-N pond was also smaller than that of the Lb-N ponds.

**Table 1:** Emission factors, seasonal emissions, and global warming potential from shrimp nursery ponds.

Nursery pond	Unit	HDPE-N pond	Lb-N pond	P–value
**Methane**				
Methane flux	mg CH_4_ m^−2^ h^−1^	0.25 ± 0.15 **b**	1.04 ± 0.75 **a**	0.004
Seasonal emission	g CH_4_ m^−2^	0.12 ± 0.05 **a**	0.50 ± 0.18 **a**	0.058
**Nitrous oxide**				
Nitrous oxide flux	μg N_2_O m^−2^ h^−1^	13.80 ± 11.26 **a**	8.37 ± 11.49 **a**	0.255
Seasonal emission	mg N_2_O m^−2^	6.62 ± 0.70 **b**	4.02 ± 0.98 **a**	0.024
**GWP**	g CO_2eq_ m^−2^	6.11 ± 1.71 **a**	18.27 ± 6.36 **a**	0.072

Lb-N pond: land-based nursery pond; HDPE-N pond: HDPE-lined pond; GWP, global warming potential; means followed by different letters within each group values (Ponds) indicates significance at P = 0.05 by Independent Samples T-Test.

The variation in the rate of N_2_O flux is displayed in Fig. 4b. The N_2_O flux during the nursing period for the HDPE-N and Lb-N ponds varied in the range of −5.63 – 23.05 μg N_2_O m^−2^ h^−1^ and −6.12 – 26.86 μg N_2_O m^−2^ h^−1^. The N_2_O flux averaged 13.8 and 8.37 μg N_2_O m^−2^ h^−1^ for the HDPE-N and Lb-N ponds, respectively (Table 1). The depletion of the HDPE-N pond showed an insignificant difference concerning N_2_O emissions rather than Lb-N ponds (P > 0.05), albeit the discrepancy was 5.43 μg N_2_O m^−2^ h^−1^ in conjunction with higher N_2_O emission detected from HDPE-N ponds. The N_2_O emission patterns of the nursery ponds were observed with higher emissions in the first periods of shrimp stocking.

Table 1 shows the GWP of the Lb-N and HDPE-N nursery ponds. The disparity in GWP emission between Lb-N and HDPE-N nursery ponds was approximately 3 folds (12.2 g CO_2_eq m^−2^), along with higher values observed in the Lb-N pond. The mean GWP's values of Lb-N and HDPE-N ponds were 18.3 g CO_2_eq m^−2^ and 6.11 g CO_2_eq m^−2^. In fact, the discrepancy was not significant (P > 0.05). CH_4_ flux was the main contributor to GWP as opposed to N_2_O flux in nursery-pond emission patterns.

### 3.2 Grow-out ponds

#### 3.2.1 Auxiliary environmental factors

**Fig. 5:**
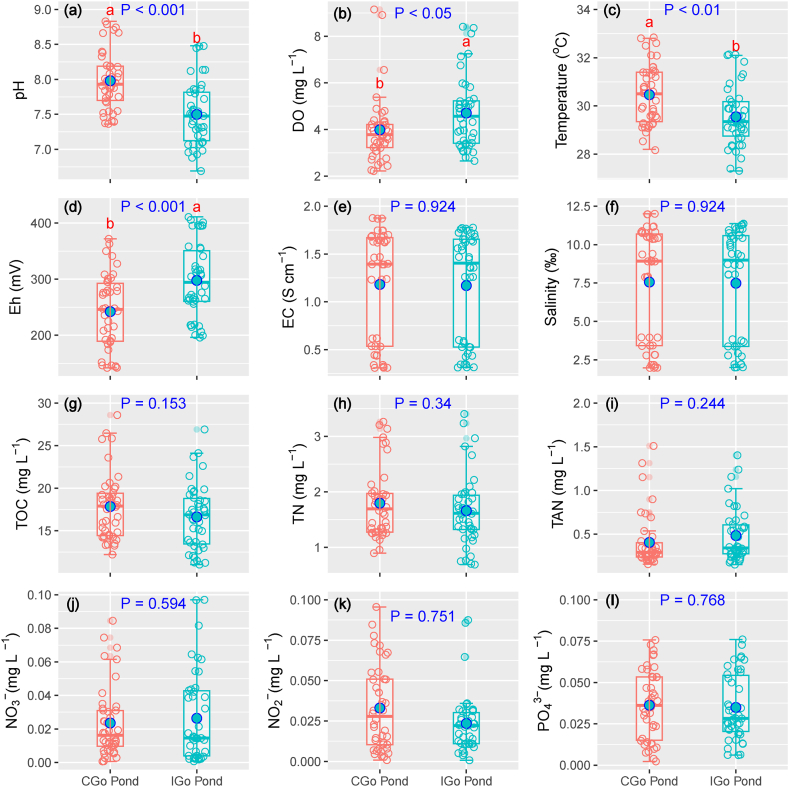
Surface water physicochemical properties in grow-out ponds. CGo pond: conventional grow-out pond; IGo pond: improved grow-out pond. Different letters in each subfigure indicate significance at P = 0.05 by Independent Samples T-Test.

The variation of environmental factors in the CGo and IGo ponds is obviously exhibited in Fig. 5. The pH value in CGo pond was higher than that of IGo pond (P < 0.001). IGo pond showed a higher DO concentration compared to CGo pond (P < 0.05). The IGO pond showed a lower temperature than CGo pond (P < 0.01). The IGo pond showed a higher Eh value than CGo pond (P < 0.001). There was insignificant in relation to EC, salinity, TOC, TN, TAN, NO_3_^−^, NO_2_^−^ and PO_4_^3−^ between grow-out ponds.

#### 3.2.2 Fluctuation in CH_4_ and N_2_O fluxes rate

**Fig. 6:**
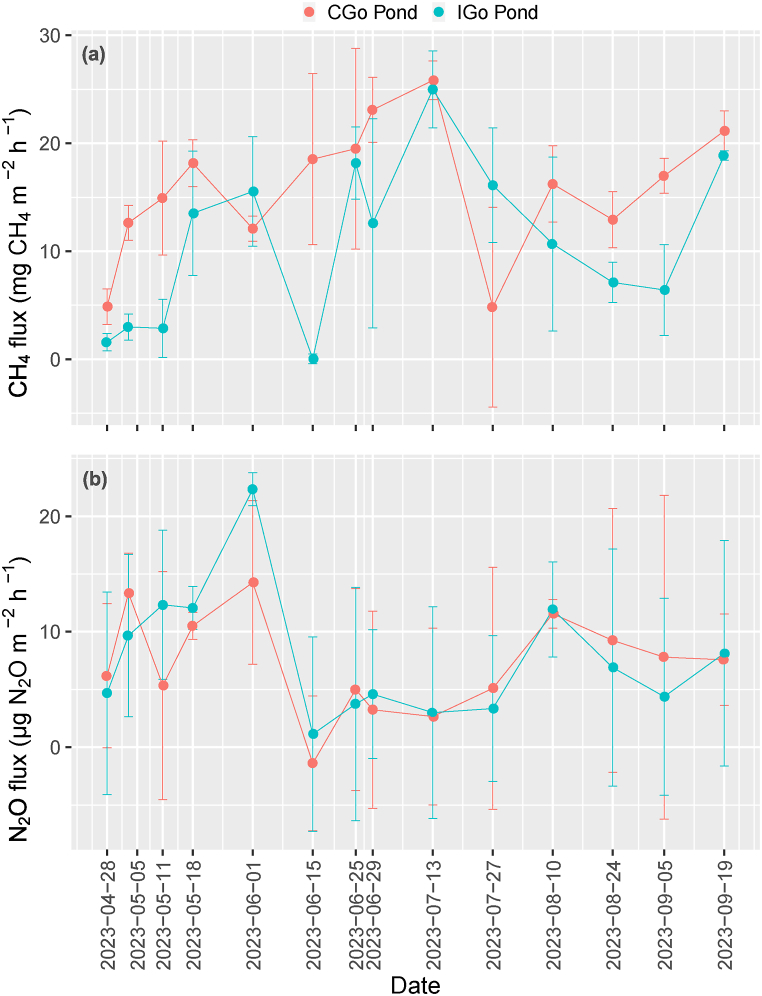
CH_4_ and N_2_O emissions from conventional and improved grow-out ponds. CGo pond: conventional grow-out pond; IGo pond: improved grow-out pond. Date format: yyyy/mm/dd.

The fluctuation of CH_4_ flux in CGo and IGo ponds varied between −5.34 – 27.5 mg CH_4_ m^−2^ h^−1^ and −0.29 – 28.0 mg CH_4_ m^−2^ h^−1,^ respectively (Fig. 6a). The CH_4_ flux pattern was complex. The highest peak was observed for both ponds at the same time. Notably, the CH_4_ flux displayed a large variation between sites and timings. Higher CH_4_ emission was revealed in the CGo ponds in contrast to IGo pond (P < 0.01) (Table 2). The mean of CH_4_ flux in the CGo and IGo ponds was 15.8 and 10.8 mg CH_4_ m^−2^ h^−1^, respectively.

**Table 2:** Emission factor, net emissions, and global warming potential from shrimp grow-out ponds.

Nursery ponds	Unit	IGo pond	CGo pond	P–value
*Methane*				
Methane flux	mg CH_4_ m^−2^ h^−1^	10.51 ± 8.50 **b**	15.84 ± 7.11 **a**	**0.003**
Seasonal emission	g CH_4_ m^−2^	47.78 ± 6.45 **a**	69.96 ± 12.30 **a**	0.069
*Nitrous oxide*				
Nitrous oxide flux	μg N_2_O m^−2^ h^−1^	7.72 ± 8.27 **a**	7.17 ± 7.86 **a**	0.752
Seasonal emission	μg N_2_O m^−2^	34.13 ± 9.73 **a**	31.67 ± 7.84 **a**	0.069
GWP	g CO_2eq_ m^−2^	1635 ± 217 **a**	2388 ± 420 **a**	0.070

CGo pond: conventional grow-out ponds; IGo pond: improved grow-out pond; means followed by different letters within each group values (Ponds) indicates significance at P = 0.05 by Independent Samples T-Test.

The variation in the N_2_O flux rate is presented in the (Fig. 6b). The N_2_O flux in the CGo and IGo ponds ranged from −6.6 – 19.0 μg N_2_O m^−2^ h^−1^ and −8.41 – 23.9 μg N_2_O m^−2^ h^−1^. High flux variation was observed during the season among ponds. The mean values of N_2_O flux in the CGo and IGo ponds were 7.17 and 7.72 μg N_2_O m^−2^ h^−1^, respectively. The N_2_O flux pattern was not significant between CGo and IGo ponds (P = 0.752) (Table 2). Similar to the CH_4_ flux, the highest peak of N_2_O flux was spotted between CGo and IGo ponds at the same time.

The GWP of CGo and IGo ponds was 2388 and 1635 g CO_2eq_ m^−2^, respectively (Table 2). The CGo pond exhibited a 1.46-fold higher GWP versus IGo pond. However, the disparity between CGo and IGo ponds was not significant (P = 0.069). The CH_4_ flux was the main source of the total GHG emission in grow-out ponds.

### 3.3 Relationship between environmental variables and GHG emissions

**Fig. 7:**
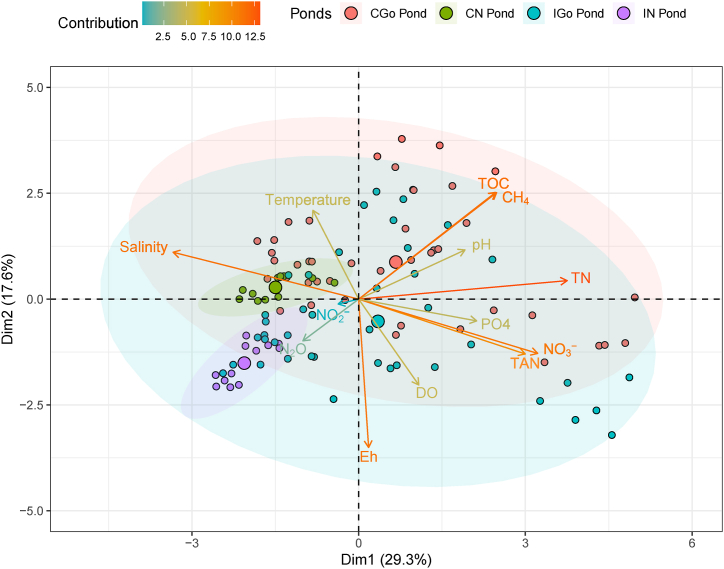
PCA of variables in relation to GHG emissions in nursery and grow-out ponds. Ellipses indicate the dispersion of data points along with principal components for each pond – larger ellipses show greater variance. Dim1 captures the most variance (29.3 % variable explanation). The change of arrow colors denotes the contributions of response variables in each dimension.

The PCA analysis conducted on nursery ponds, both environmental factors, and CH_4_ and N_2_O flux rates indicated that most of the analysed variables were interdependent and significantly intercorrelated with 13 Dims (Figs. S4–S5). Dim1 and Dim2 were significant for observing the relationship between environmental variables and GHG-emission factors. The explanation of the two components was extracted by 29.3 % (Dim1) and 17.6 % (Dim2), which contributed a total of 46.9 % of variables (Fig. 7). The first component (Dim1) pointed out the interdependence among salinity, TOC, CH_4_, TN, TAN, and NO_3_^−^ which contributed 16.4, 9.1, 8.9, 20.7, 13.2and 15.3 % to the Dim1, respectively. The second component showed a connective intercorrelation among DO, temperature, Eh, TOC, and CH_4_, accounting for 10.1, 10.8, 30.1, 15.5 and 15.6 % to the Dim2.

**Fig. 8:**
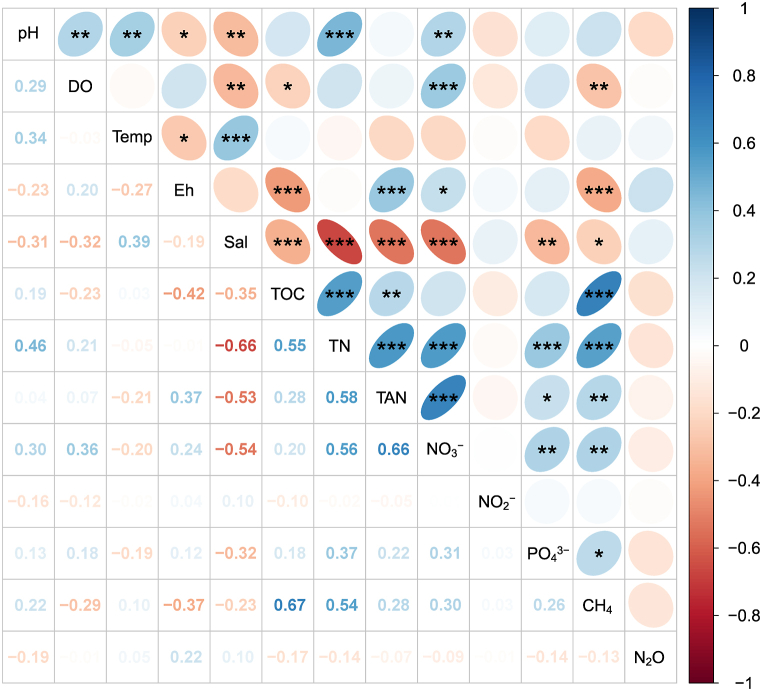
Pearson correlation between variables in nursery and grow-out ponds. The value in grids is the Pearson correlation coefficient (r), also marked by colors. *P < 0.05, **P < 0.01 and ***P < 0.001. Temp., temperature; Sal., salinity.

**Fig. 9:**
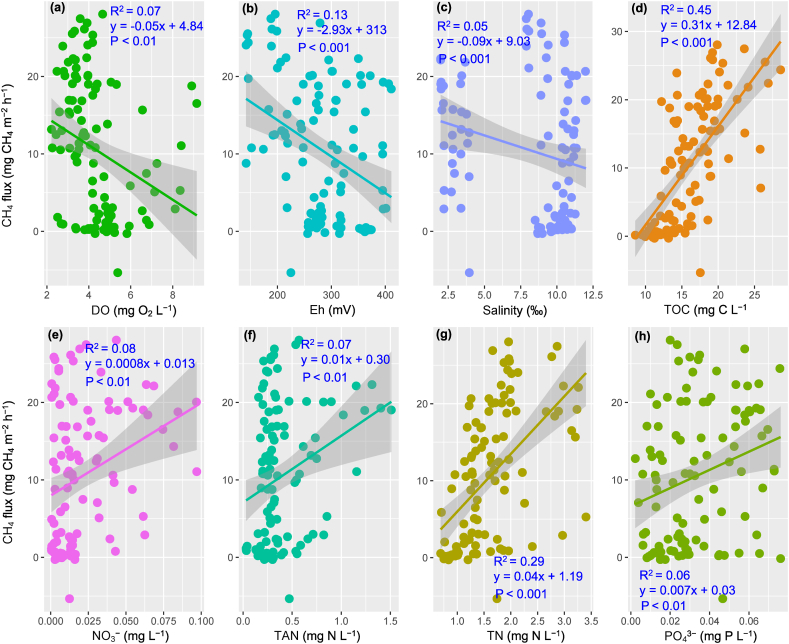
Linear models of the relationship between CH_4_ flux versus TOC, TN, DO and Eh.

Pearson analysis illustrated the relationship between environmental variables and its connection to GHG emissions, comprising DO, Eh, salinity, TOC, TN, TAN, NO_3_^−^, and PO_4_^3−^ (Fig. 8). The strongest correlation was identified between CH_4_ flux and concentrations of TOC (r = 0.67), and TN (r = 0.54). Linear models of these relationships are displayed in Fig. 9a–h. The positive relationship between TOC and CH_4_ was clearly observed (R^2^ = 0.45), while the other showed low R^2^ values (<0.29), although the relationship was significant in both the Pearson test and linear regression (P < 0.05). It was discovered that there was an uncorrelation between response variables and N_2_O flux (r < 0.50).

Based on PCA and Pearson correlations, the variance of CH_4_ flux could be controlled by DO, temperature, Eh, salinity, TOC, TN, TAN, and NO_3_^−^. As such, a GLM model was fitted to predict the relationship between vital factors contributing to the CH_4_ flux in aquaculture pond systems (Table 3). The effects of DO, Eh, salinity, TOC, TN and NO_3_^−^ were significant in the predicted models. The model's explanatory power was substantial (R^2^ = 0.62).

**Table 3:** Generalised linear regression between response variables and CH_4_ emission.

Predictors	Estimates	CI	P-value
(Intercept)	−5.01	−28.52–18.50	0.676
DO	−2.06***	−3.02–−1.11	**<0.001**
Temp	0.33	−0.40–1.07	0.373
Eh	−0.02*	−0.05–−0.00	**0.021**
Sal	0.18	−0.34–0.70	0.496
TOC	0.63**	0.22–1.03	**0.002**
TN	5.01***	2.12–7.89	**0.001**
TAN	−1.74	−7.86–4.37	0.577
NO_3_^−^	116.96**	44.00–189.92	**0.002**

Observation, 108; R^2^ = 0.623; *p < 0.05 **p < 0.01 ***p < 0.001; 95 % confidence intervals (CIs) and p-values were computed using a Wald t-distribution approximation.

## 4 Discussion

### 4.1 Variation of environmental parameters in nursery ponds

The application of nursery ponds is necessary to minimise adverse environmental factors to shrimp growth as shrimp gradually adapt to the on-site environment as well as being eradicated by predators in aquatic ecosystems [31]. Shrimp larvae are generally nurtured in a local hatchery that optimises nutrients and environmental conditions. If shrimp larvae are stocked in grow-out ponds directly, environmentally initial stress/shock is the primary cause of increasing mortality and reducing shrimp-harvested productivity. The suggestion of a 20-day stage in a nursery pond was ideally to improve technical procedures and control suitable shrimp density when considering developing the sustainable rice-shrimp rotation system [10].

The results indicated that the application of HDPE-lined nursery ponds shows a slight variation in environmental parameters, including DO, temperature, Eh, EC, TAN, and TN, rather than land-based nursery ponds, albeit the density was comparable (Fig. 3). The temperature constancy could be explained by covering a net for the HDPE-lined pond that reduces light intensity, diminishing the rapid shift of water temperature. Higher Eh in the HDPE-lined pond caused by the operation of continuous aeration systems. Although there was an insignificant difference among TAN, NO_3_^−^, NO_2_^−^, and PO_4_^3−^ between the Lb-N and HDPE-lined pond, the Lb-N pond still showed a high-concentration trend compared to the HDPE-lined nursery pond. Discharging bottom wastewater and supplying a clean water source for HDPE-lined nursery ponds could partly eliminate TOC and TN pollution and ameliorate a metabolic equilibrium status compared to the land-based nursery pond. Without releasing shrimp, and wastewater would store the organic matter and nutrients [12]. Moreover, the Lb-N ponds maintained a stagnant condition, which could allow a better accumulation of TOC and nutrients [12]. The environmental stability in the HDPE-lined nursery pond could maintain an appropriate environment for the growth of shrimp larvae.

### 4.2 Variation of environmental parameters in nursery and grow-out ponds

The large variation of environmental factors was disclosed in the grow-out ponds via timings (Fig. 5). DO values improved substantially in IGo pond, indicating improved environmental quality [32]. Level of DO from 4 to 5 mg L^−1^ or higher optimises for aquaculture ponds [33]. Low DO concentration influences the growth and productivity of aquaculture ponds [34]. This study recorded several times that the DO values were below the ideal range, especially in the CGo pond. Likewise, an improvement of DO for the grow-out pond is required. Higher Eh value in the IGo ponds was relative to the advancement of DO concentration. The variation of EC and salinity was significant between CGo and IGo ponds. Also, the downward trend of the EC and salinity values was gradually reliable. One possible explanation was the transition between the dry and wet seasons. The adoption of rainfed was caused by reducing EC and salinity in the grow-out ponds [35]. The environmental parameters of TOC, TAN, NO_3_^−^, NO_2_^−^, and PO_4_^3−^ were insignificant between ponds, but the change was considerable between timings, indicating temporal variation rather than spatial alteration. The dynamic of these variables was reported in the grow-out ponds of the rice-shrimp rotation system, *P. vannamei* and *P. monodon* ponds of the VMD [7,35]. This study revealed that the variation of TOC concentration was outlined over the range from previous studies in various timings, while TAN, NO_3_^−^, NO_2_^−^, and PO_4_^3−^ were ranged between the mentioned studies.

### 4.3 Factors affecting CH_4_ and N_2_O emissions from aquaculture ponds

Emissions from shrimp nursery ponds are also rare from previous reports, especially in the VMD. This study provided the CH_4_ and N_2_O emission factors for conventional and HDPE-lined nursery ponds for shrimp, a typical model applied in the VMD. It was evident that applying HDPE-lined nursery ponds with continuous aeration could reduce CH_4_ flux, but it stimulates the N_2_O flux from that pond compared to land-based nursery ponds [36]. Although the aeration process was executed continuously for HDPE-N ponds, DO values were lower than that of Lb-N ponds. This could be explained by the fact that the shrimp density stocked in the HDPE-N ponds was 2.5 folds higher than that of the Lb-N pond. The lower DO definitely favours methanogens that trigger CH_4_ flux in HDPE-N ponds [37,38]. In contrast, high DO content restrains CH_4_ generation in the soil as well as CH_4_ oxidation in the water column [39]. However, the flux in the HDPE-N pond did not include the emission from wastewater components removed from the bottom of HDPE pond as well as electrical energy consumption for carrying aeration. It is suggested that emission factor from that point should be noted for further research.

Aquaculture ponds are known to have CH_4_ emissions than lakes, rivers, and reservoirs as high organic loadings from feeds and animal wastes [12]. In the VMD's shrimp-crab co-culture, farms take advantage of rice stubble from preceding crops as a source of nature-based feeds for shrimp-crab. The residual rice-stubble biomass utilised for shrimp-crab ponds was a reliable carbon source in the systems. The submerging of the biomass donates essential substrates for methanogenesis [15]. This study found a positive relationship between CH_4_ and TOC, indicating a carbon source contributor to CH_4_ flux [40]. It was found that the return of straw in aquaculture ponds increased CH_4_ emission by 35–46 % compared to straw removal in integrated rice-crayfish farming [41]. This study disclosed that the magnitude of TN affected the CH_4_ flux, inferring the increasing TN in shrimp-cab co-culture caused CH_4_ flux augmentation as nitrogen is also a vital nutrient for microorganisms. However, nitrogen's effects on methanogen activity were complex, as highlighted by several studies [42]. Previous studies displayed that the nitrogen factor could stimulate methanogen activities or decrease the methanogen community in various environments [43,44]. The findings indicated that CH_4_ flux negatively correlated to DO and Eh values. As higher DO and Eh values shrank CH_4_ emissions in the shrimp ponds, suggesting the reduction of methanogenesis and enhancement of methanotrophy [13].

The N_2_O emission from the aquaculture ponds showed a complex mechanism as incomplete denitrification and nitrification affected by water column DO concentration [11,13]. This study illustrated that the relationship between environmental parameters and N_2_O emission was negligible. This could be attributed to the insignificant variation of nutrients (TAN, NO_3_^−^, NO_2_^−^ and PO_4_^3−^) in the shrimp-crab co-culture ponds (Fig. 3; Fig. 5). However, several studies demonstrated that N_2_O emission in intensive shrimp ponds was relative to nutrients. For example, Li et al. [11] revealed the correlation between N_2_O flux and TN, as well as NH_4_^+^ and NO_2_^−^. Likewise, Yang et al. [16] revealed a relationship among N_2_O emission versus pH, DO, salinity and NH_4_^+^.

One of the possible reasons was hypothesised that no external feed was applied during the culture. Grow-out ponds were run based on the equilibrium of nature. In fact, the availability of NO_3_^−^, and NH_4_^+^ was subjected to a nitrification and denitrification process that increased N_2_O flux in aquaculture ponds [17]. Therefore, N_2_O could generated by microorganisms under aerobic and anaerobic conditions [[45], [46], [47], [48]].

Various studies have pointed out emission pathways from aquaculture ponds [13,17,49]. Ebullition and diffusion are vital mechanisms of GHG emissions in aquaculture ponds. Ebullition may strongly contribute to the overall CH_4_ flux by 75–96 % [13]. In fact, CH_4_ was indeed less soluble than N_2_O. Therefore, the contribution of CH_4_ emission to total GHG emissions was substantial, which is consistent with the current study. Several studies investigated GHG emissions from crab-fish, rice-fish, rice-crayfish, rice-crab, crab-fish, shrimp, shrimp-fish, and crab aquaculture systems from various countries. Methane flux varied from 0.37 to 37 mg CH_4_ m^−2^ h^−1^, while N_2_O emission ranged between 9.76 and 48.1 μg CH_4_ m^−2^ h^−1^ [11,16,25,40,[50], [51], [52], [53], [54], [55], [56]] (Table S3). The current study found that the variation of CH_4_ and N_2_O emissions was comparable with previous reports.

### 4.4 Moving-forward strategies reducing CH_4_ and N_2_O emissions from shrimp-crab cocultured ponds

The current investigation showed that the decisive discrepancies of CGo and IGo ponds comprised *(i)* stocking shrimp three times in the CGo ponds (2.1 shrimp m^−2^) greater than IGo ponds two times (3.1 shrimp m^−2^), *(ii)* higher mud crab density in CGo pond (13.4 crab m^−2^) than IGo pond (0.15 crab m^−2^), *(iii)* frequency of bioproduct application in the IGo pond (7 times) beyond the CGo pond (2 times). The inconsistency of the density of shrimp and crab was challenging for the explanation of environmental variation throughout the current study. However, several studies illustrated that higher density triggered a reduction of DO concentration in water bodies [57,58], while others identified the role of shrimp, fish, and crab could reduce bubble built up via bioturbation [59]. Hence, co-culture objects in aquaculture ponds are crucial in enlarging or shrinking GHG emissions.

This study noticed that intensive bioproduct application (∼164 L ha^−1^) would improve the DO concentration for the grow-out pond, resulting in CH_4_ flux reduction in the IGo pond. The EM bioproduct positively affects DO improvement and biological oxygen demand (BOD) reductions [7,60]. The increase of DO in ponds could greatly contribute to the decrease of overall GHG emissions as CH_4_ is the major contributor to emissions in aquaculture ponds [13,15,40]. However, the remaining rice stubbles in the system are one of the major challenges in developing lower-GHG emission shrimp-crab co-culture production. The application of bioproducts is a cheaper, simpler, and environmentally friendly method to ameliorate lowering GHG emissions. However, this study has not provided enough evidence concerning appropriate doses or frequencies of EM bioproducts for reducing GHG emissions. Hence, we recommended the optimisation of EM-applied bioproduct doses and frequencies to improve DO concentration and quantify the amount of GHG emission reduction in the models.

Keeping rice stubble on the platform is a practical recommended technology when it comes to providing natural feeds for shrimp-crab co-culture. However, rice stubble height remained in the forum, or on the other hand, the amount of rice biomass on the platform is a key carbon source for the decomposition under waterlogging, resulting in higher labile carbon in the water column. This study revealed a positive relationship between TOC and CH_4_ flux in shrimp-crab co-culture ponds when the rice stubble height was 10 cm from the platform. Thus, the relationship among rice stubble, TOC and CH_4_ needs to be examined for further research to clarify the GHG emission patterns and have appropriate guidelines for developing rice-shrimp rotation systems in the VMD. Additionally, intensification of CO_2_ uptake from phytoplankton or growing aquatic fast-biomass plants could be a promising long-term solution to offset GHG emissions from CH_4_ and N_2_O emissions in aquaculture ponds.

This study also recorded fewer water-exchange frequencies (Table S2), resulting in a low DO concentration. Clean water exchange regularity or supplementation probiotics that contain *lactobacillus* and *bacillus* ssp. is essential to renewing and improving water quality in production ponds [7]. Moreover, dredging sediment preceding shrimp stock could potentially boost water quality.

Mixed species are also considered an optional solution to reduce overall GHG emissions. High stocking density of one species in aquaculture ponds would deplete DO concentration [57]. Zhang et al. [12] demonstrate that shrimp monoculture had much CH_4_ flux rather than shrimp-crab co-culture. This result was consistent with Fang et al. [51] and Yang et al. [16], who hypothesised that mixed species effectively utilised the organic matter in the water column as well as donated biomethanation substrates. Moreover, the combination of bivalves in the rotation systems could be feasible for assimilating dissolved organic carbon as well as consuming faeces, pseudofeces or excretion of species in aquaculture ponds [61]. Improved water quality would moderate carbon substrates for methanogens that reduce methane emissions.

## 5 Conclusions

This study examined CH_4_ and N_2_O emissions from Lb-N and HDPE-lined nursery ponds and CGo and IGo models in rice-shrimp rotation systems. Land-based nursery ponds emitted higher CH_4_ flux and lower N_2_O emission compared to HDPE-lined nursery ponds. The GWP of Lb-N ponds was 3-fold higher than that of the HDPE-lined nursery ponds. The variation in GHG emissions was obviously caused by temporal levels rather than spatial magnitude. The IGo pond had a lower CH_4_ flux factor than the CGo pond, while N_2_O emission was insignificant. Subsequently, the GWP of the CGo pond was 1.5 times higher than the emission of the IGo models. Applying land-based nursery ponds combined with conventional grow-out ponds can cause higher GHG emissions and GWP. This study underlined that a high frequency of co-incubated inoculum could improve DO concentration and reduce CH_4_ emission. The availability of TOC concentration in aquaculture ponds was also a good indicator for the enlargement of CH_4_ emission. This study suggests further work, including *(i)* clarifying the relationship between rice stubble remainder on the platform and TOC, as well as GHG emissions, and (ii) improving DO concentration in grow-out ponds, including EM probiotics, water exchange, bivalves, aquatic plants, and mixed species in the aquaculture system to improve water environmental quality and offset GHG emissions.

## Data availability

The data that support this study are available from the corresponding author upon reasonable request.

## CRediT authorship contribution statement

**Huynh Van Thao:** Writing – review & editing, Writing – original draft, Visualization, Methodology, Investigation, Funding acquisition, Formal analysis, Data curation, Conceptualization. **Nguyen Van Cong:** Writing – review & editing, Validation, Supervision, Methodology, Conceptualization. **Le Thi Cam Nhung:** Writing – review & editing, Visualization, Software, Formal analysis. **Tran Hoang Kha:** Writing – review & editing, Investigation, Formal analysis, Data curation. **Huynh Cong Khanh:** Writing – review & editing, Software, Investigation. **Le Van Dang:** Writing – review & editing, Software, Methodology. **Nguyen Phuong Duy:** Writing – review & editing, Project administration, Methodology, Investigation. **Huynh Quoc Tinh:** Writing – review & editing, Project administration, Methodology, Investigation, Conceptualization. **Trieu Nguyen Lan Vi:** Writing – review & editing, Project administration, Methodology, Investigation. **Nguyen Phuong Chi:** Writing – review & editing, Investigation, Formal analysis, Data curation. **Tran Sy Nam:** Writing – review & editing, Validation, Supervision, Methodology, Investigation, Formal analysis, Data curation, Conceptualization.

## Declaration of competing interest

The authors declare that they have no known competing financial interests or personal relationships that could have appeared to influence the work reported in this paper.
