# Supplementary material for: Methane and nitrous oxide emissions in the rice-shrimp rotation system of the Vietnamese Mekong Delta
Source: Heliyon. 2024 Aug 8;10(16):e35759. doi: 10.1016/j.heliyon.2024.e35759 (PMC11379990; doi:10.1016/j.heliyon.2024.e35759)
Supplement: Multimedia component 1 [file mmc1.docx]

**Supplementary figure**


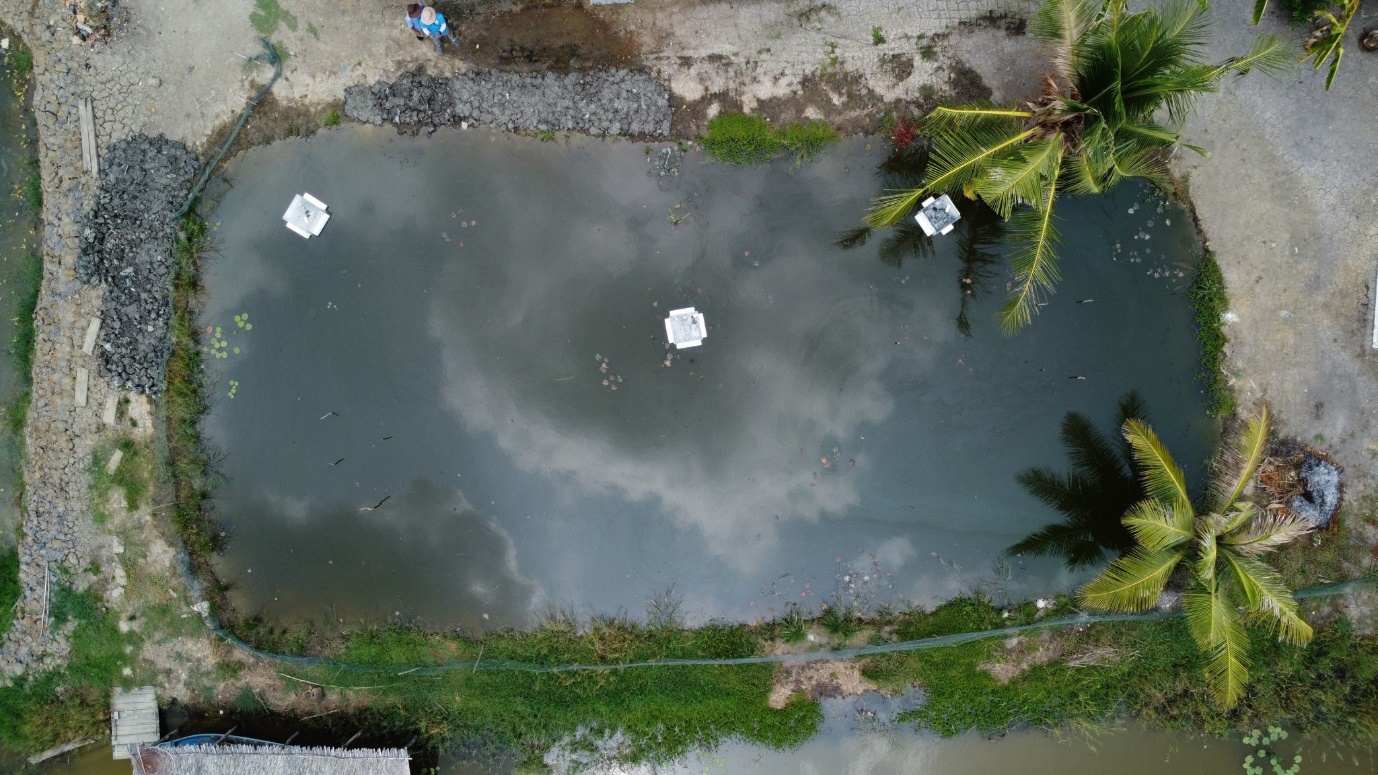

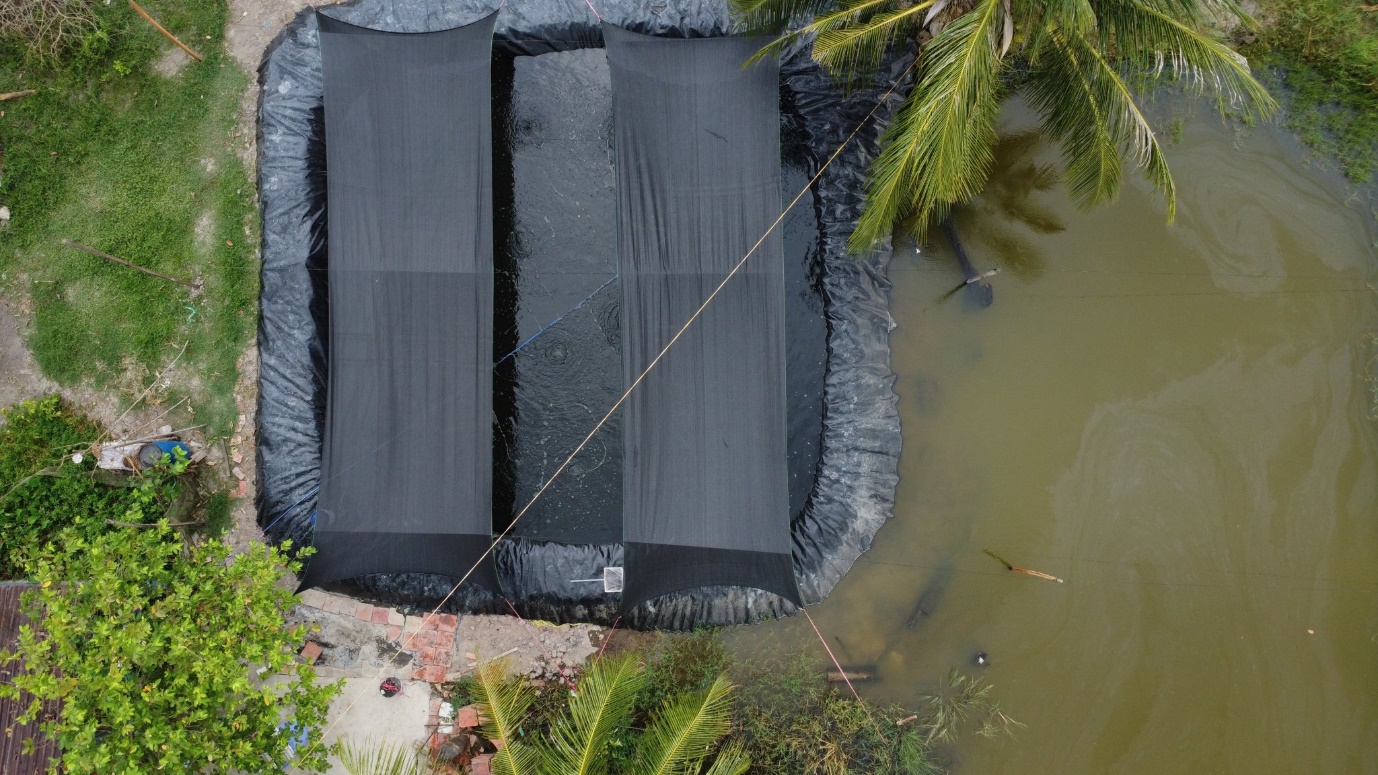


**(a)**

**(b)**

**Fig**. **S1. Land-based nursery pond (a) and HDPE-lined** **pond (b)**


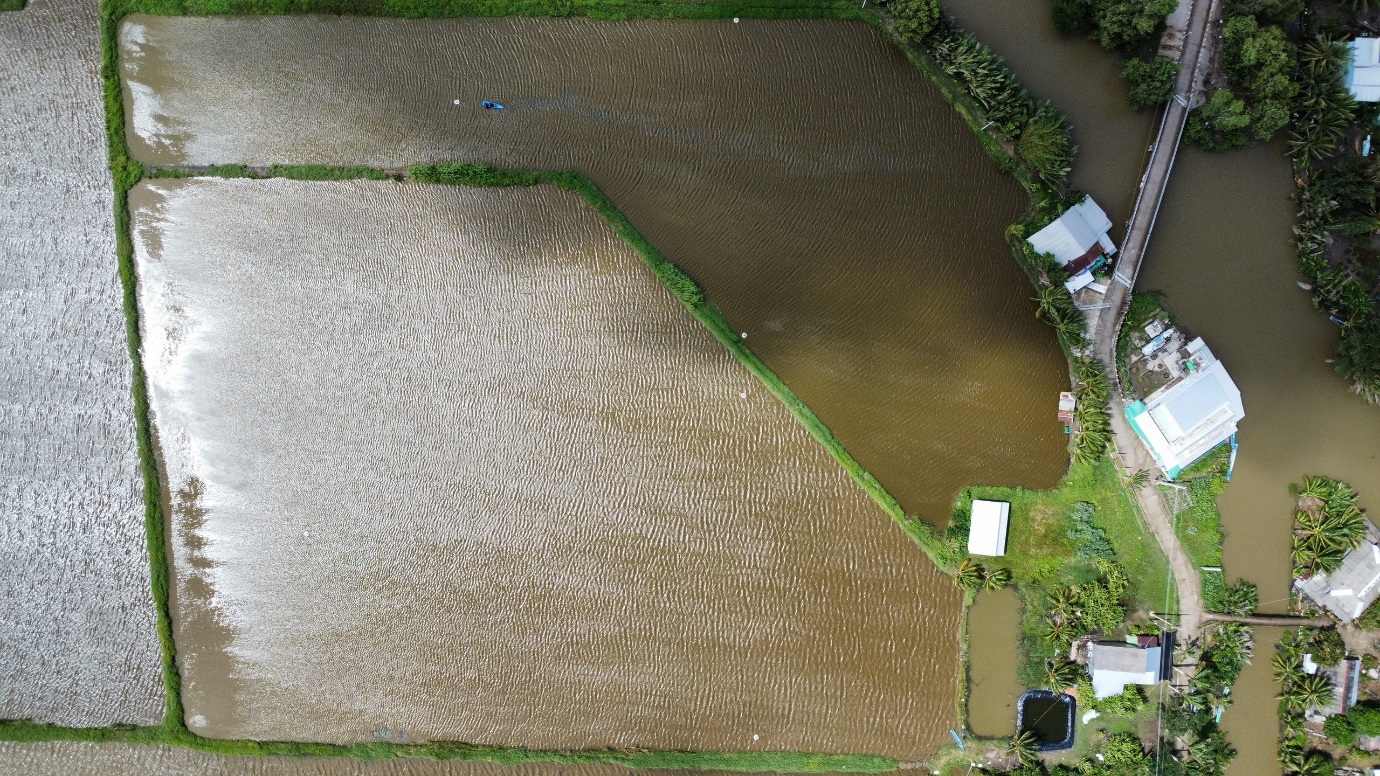


**(a)**

**(b)**

**Fig**. **S2. Conventional grow-out ponds (a) and improved grow-out pond (b) in the rice-shrimp rotation systems**


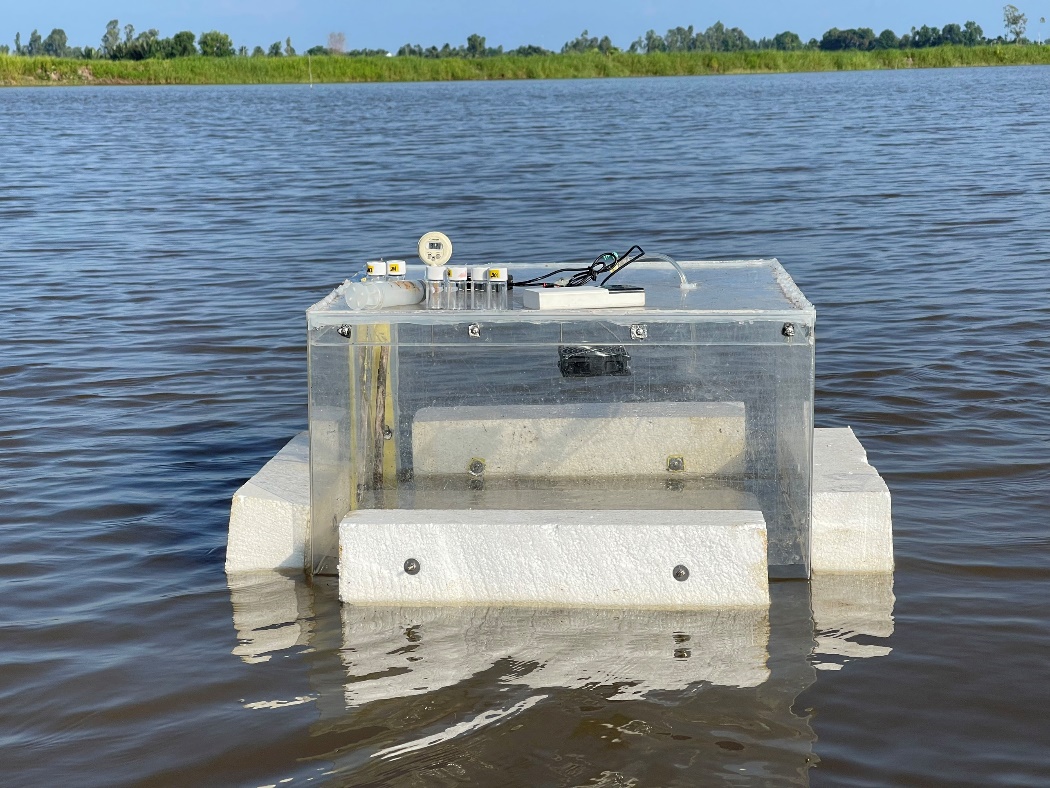


**Fig**. **S3. The deployment of floating chamber to collect gas samples on aquaculture ponds**

**
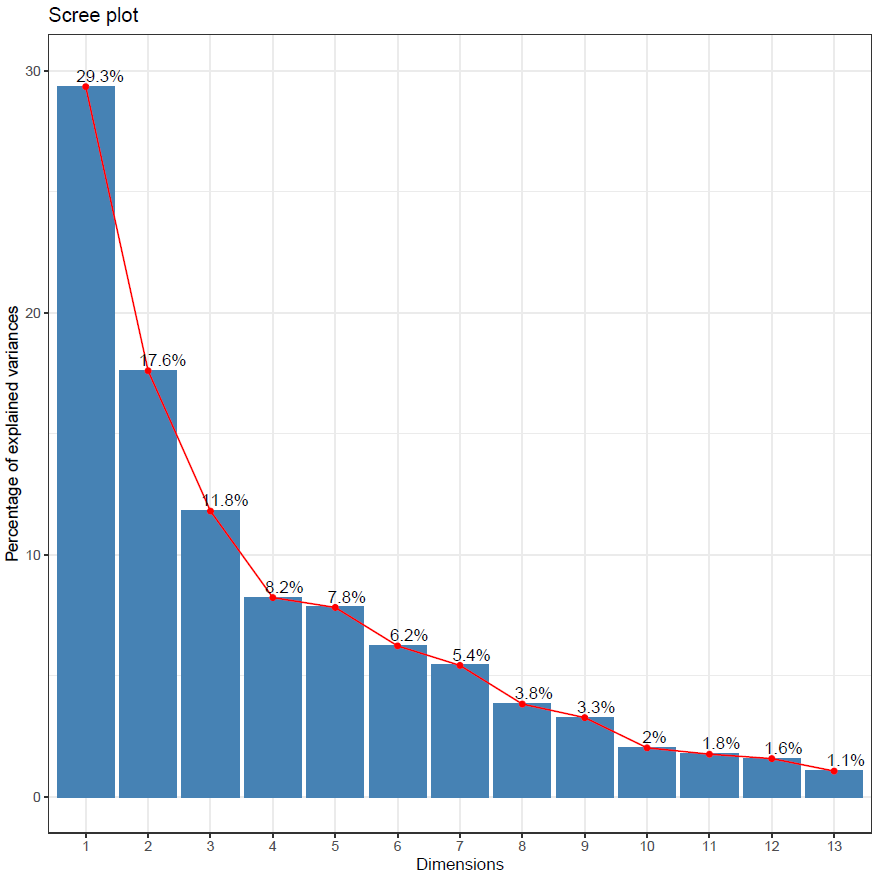
**

**Fig. S4. Screeplot shows the percentage contribution of dimmensions in PCA.**

**
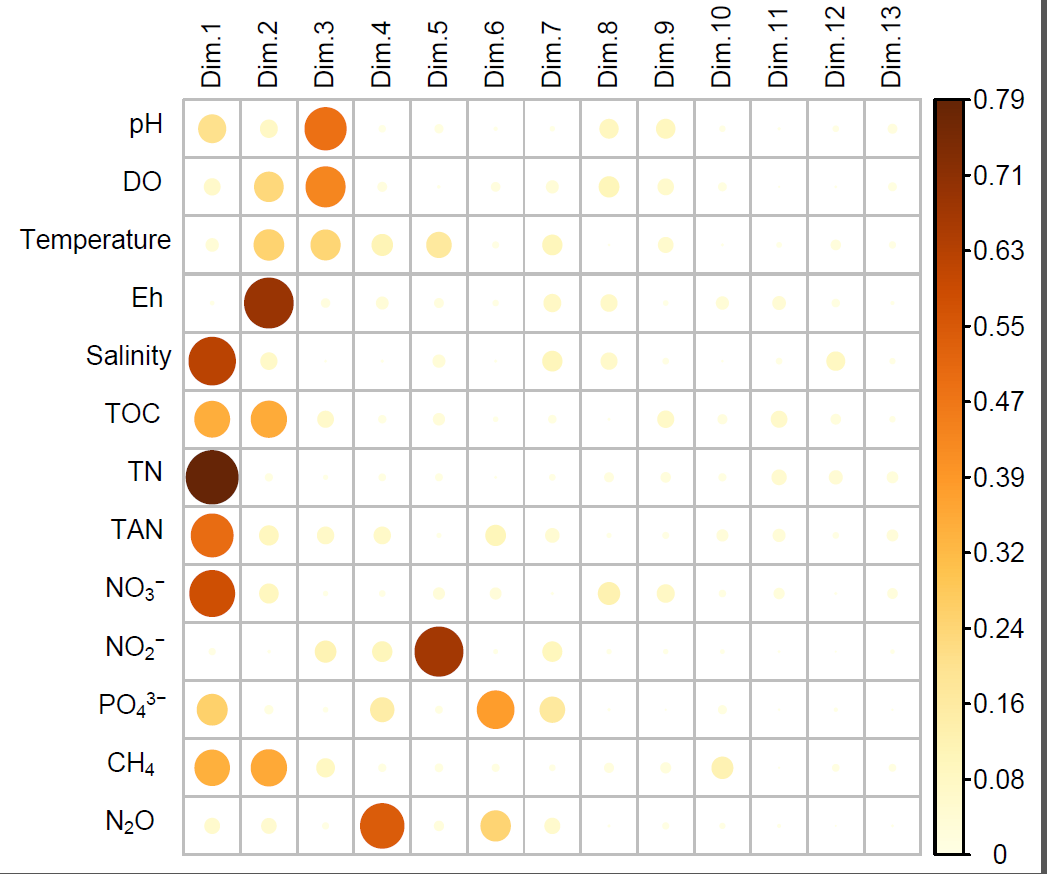
**

**Figure S5. Contribution of variables in each dimmension in PCA.**
