# Supplementary material for: Methane and nitrous oxide emissions in the rice-shrimp rotation system of the Vietnamese Mekong Delta
Source: Heliyon. 2024 Aug 8;10(16):e35759. doi: 10.1016/j.heliyon.2024.e35759 (PMC11379990; doi:10.1016/j.heliyon.2024.e35759)
Supplement: Multimedia component 2 [file mmc2.docx]

**Supplementary Tables**

**Table S1. General information and farming practices in the nursery ponds**

| Items | Unit | Lb-N pond | HDPE-N pond |
| --- | --- | --- | --- |
| Area | m^2^ | 150 | 80 |
| Depth | m | 1.5 | 0.8 |
| Nurturing period | day | 19 | 19 |
| Aeration | h d^–1^ | NA | 24 |
| Shrimp waste removal | time d^–1^ | NA | 3 |
| Co-incubated inoculum | day | 3 | 3 |
| Shrimp density | shrimp m^–2^ | *160*  *(28/04 – 17/05/23) ^†^* | *400*  *(28/04 – 14/05/23) ^†^* |

Note: “^†^” date format: dd/mm/yy

**Table S2. General information and farming practices in the grow-out ponds**

| **Practices** | **Unit** | **Conventional pond** | **Improved pond** |
| --- | --- | --- | --- |
| Total area | m^2^ | 9,186 | 15,335 |
| *Ditch area* | m^2^ | 2,094 | 2,680 |
| *Platform* | m^2^ | 6,632 | 11,935 |
| *Embankment* | m^–2^ | 460 | 720 |
| Water depth |  |  |  |
| *Ditch* | cm | 80 – 120 | 80 – 120 |
| *Platform* | cm | 10 – 45 | 10 – 45 |
| Farm preparation |  |  |  |
| *Liming* | kg ha^–1^ | 85.9 | 85.6 |
| *Flooding platform* | cm | 20 – 30 (09– 12/01/23)  20 – 30 (23 – 26/01/23)  40 (06 – 09/02/23) | 20 – 30 (16 – 20/01/23)  40 (01 – 04/02/23 |
| *Fertilising* | kg ha^–1^ | 57^†^ (15/02/23) | 52^‡^ (14/02/23) |
| Stocking shrimp | shrimp m^–2^ | 0.57 (28/02/23) | 2.46 (10/03/23) |
|  |  | 0.57 (29/03/23) | 1.64 (28/4/2023) |
|  |  | 1.14 (28/04/23) | 0.82 (02/06/23) |
|  |  | 1.03 (29/05/23) |  |
|  |  | 1.15 (28/06/23) |  |
| Stocking mud crab | crab m^–2^ | 0.06 (09/04/23)  0.20 (05/05/23) | 0.15 (04/04/23) |
| Water exchange | % | –80 (21/6/23)  80 (28/06/23)  –60 (16/8/23)  60 (22/08/23) | –80 (21/6/23)  80 (28/06/23)  –60 (16/08/23)  60 (22/08/23) |
| Fertilisating | kg ha^–1^ | 57^†^ (20/03/23) | 27.4^‡^ (03/06/23) |
|  |  | 57^†^ (20/03/23) | 27.4^‡^ (10/06/23) |
| Final harvest |  | (19/09/23) | (19/09/23) |

Note: “^†^” applying chemical fertiliser that contains 16% N kg^–1^, 45% P_2_O_5_ kg^–1^ and 2.5% K_2_O kg^–1^; “^‡^” applying organic fertiliser (commercial name: Trùn Đỏ) that contain Moisture, 10%; SiO_2_,0.63 – 0.65 g kg^–1^;CaCO_3_; 5 – 7 g kg^–1^; MgSO_4_, 8 – 10 g kg^–1^; FeSO_4_, 50 – 70 g kg^–1^. The value in parenthesis indicates the date of application (date format: dd/mm/yy)

**Table S3. Comparison of CH_4_ and N_2_O emissions from aquaculture ponds among countries**

| **Pond** | **Location** | **CH_4_ flux**  **mg m^−2^ hr^−1^** | **N_2_O flux**  **µg m^−2^ hr^−1^** | **References** |
| --- | --- | --- | --- | --- |
| Crab-fish | Xinghua, China | 0.50 – 1.14 | NA | Hu et al., (2016) |
| Rice–fish | Cuttack, India | 2.48 – 2.52 | 29.57 – 29.77 | Datta et al., (2009) |
| Rice-crab/fish | Stuttgart, Germany | 13.6 – 20.0 | NA | Frei and Becker, (2005) |
| Rice–fish | Mymensingh, Bangladesh | 32 - 37 | NA | Frei et al. (2007) |
| Rice-crayfish | China | 4.59 | 39.5 | Fang et al., (2023) |
| Rice-crab | Panjin, China | 11.57 – 12.33 | 21.1 - 27 | Wang et al., (2019) |
| Crab-fish | Southeast, China | 0.37 | 48.1 | Liu et al., (2016) |
| Shrimp | Zhejiang, China | NA | 9.76 | Li et al., (2019) |
| Shrimp  Shrimp-fish | Shanyutan, China | 19.55  1.65 | 10.74  11.8 | Yang et al. (2015) |
| Crab | Jiangsu, China | 0.54 – 0.61 | 34.2 – 34.8 | Ma et al. (2018) |
| Aquaculture | Shanyutan, China | 17.4 | NA | Yang et al. (2022) |
| Shrimp^‡^ | Mekong Delta, Vietnam | 0.29 – 1.39 | 6.05 – 15.48 | **Current study** |
| Shrimp-crab ^†^ | Mekong Delta, Vietnam | 9.15 – 18.06 | 5.34 – 9.71 | **Current study** |

*NA: non-applicable; ^‡^nursery pond for land-based and HDPE-Lined pond with aeration operated continuously; ^†^grow-out ponds in rice-shrimp rotation for improved and conventional practices.*
